# Harnessing lived experience in health professions simulation-based education: a scoping review

**DOI:** 10.1007/s10459-025-10432-9

**Published:** 2025-04-24

**Authors:** Renee Molloy, James Bonnamy, Gabrielle Brand, Nicole Pope, Richard Schweizer, Samantha Sevenhuysen

**Affiliations:** 1 School of Nursing and Midwifery, Sub-Faculty of Health Sciences, Faculty of Medicine, Nursing and Health Sciences, Monash University, Victoria, Australia; 2 Department of Nursing, Melbourne School of Health Sciences, Faculty of Medicine, Dentistry, and Health Sciences, The University of Melbourne, Melbourne, VIC Australia; 3 Murdoch Children’s Research Institute, and Royal Children’s Hospital, Melbourne, VIC Australia; 4 Child Health Evaluative Services, Research Institute, The Hospital for Sick Children, Toronto, Canada; 5 Western Australian Group for Evidence Informed Healthcare Practice: A JBI Centre of Excellence, Curtin University, Perth, WA Australia; 6 One Door Mental Health, Paramatta, NSW Australia; 7 Peninsula Health, Frankston, VIC Australia

**Keywords:** Simulation-based education, Lived experience, Health professions education

## Abstract

Simulation is an established pedagogical approach in health professions education, typically led by educators and informed by their clinical expertise. Partnerships between educators and people with lived experience ensures simulation authentically represents the needs of people accessing healthcare. To map available literature on how lived experiences are incorporated into health professions simulation-based education a scoping review was conducted. In April 2024 CINAHL Complete, Scopus, ERIC, Medline, PsycINFO, and ProQuest Dissertations and Theses Global Database were searched. Studies were screened against the inclusion criteria, and data was extracted from 45 studies using a purposively developed and piloted extraction tool, and organised according to four research questions. Medicine and nursing most commonly include lived experiences in simulation-based education and cultural and linguistic diversity is the lived experience most often harnessed. Lived experience involvement across the entire six phases of simulation design and delivery was not common, however active and meaningful involvement was represented at each stage. Lived experience involvement enhances simulation-based education and provides an additional opportunity for people with lived experience to be involved in health professions education. There is an urgent need for guidelines describing how educators can harness lived experiences in simulation-based education. Further research, in partnership with people with lived experience, is required to determine how to more authentically represent lived experience in simulation-based education.

**Supplementary Information:**

The online version contains supplementary material available at 10.1007/s10459-025-10432-9.

## Introduction

Key Words: Simulation-based education, lived experience, health professions education

The active involvement of real people and their unique experiences of health and healthcare (lived experience) is now widely considered best practice in research, education, healthcare delivery and quality improvement (Baines & Regan de Bere, 2018). Accreditation bodies for health professions education (HPE) increasingly require inclusion of lived experiences (LE) in curricula (Fossey et al., 2023), challenging traditional notions of privileging the knowledge of academics, educators and clinicians over the knowledges of people with lived and living experiences (Brand & Dart, 2022). This approach offers empowerment, healing, and emancipation for people sharing their lived experiences (Kangasjarvi et al., 2023), and learners find it engaging, transformative, and an opportunity for reflexive learning (Eijkelboom et al., 2023; Massé et al., 2023). Integrating LE into HPE has been shown to improve learners’ communication skills, attitudes and empathy, and deepen their understanding of the needs of people who access healthcare (Massé et al., 2023; Symon et al., 2021).

It has been suggested that partnerships with people with LE should be central to the education of future health professionals (Eijkelboom et al., 2023). Yet, despite increasing calls from health professional accreditation bodies, policymakers, and people with LE to increase active participation and partnerships in HPE, aspirations are ahead of practice (Molloy et al., 2023). There is no consensus on what partnership means for all stakeholders or how this can be implemented in practice (Bennett-Weston et al., 2024). The integration of LE into HPE is often categorised across a spectrum, from no involvement (e.g., teaching and learning planned and delivered without LE consultation or involvement) to full partnership (e.g., people with LE are employed as consumer academics to plan and deliver education) (Tew et al., 2004). However, recent findings from a qualitative study have challenged the notion of partnerships only being achievable at the highest level of involvement, instead suggesting that true partnerships are about valuing people with LE for their contributions, regardless of the level of involvement (Bennett-Weston et al., 2024). One-off guest lectures, or ‘teaching’ as it is commonly referred to, is the most prominent area of LE involvement cited in a recent health professions HPE literature review (Soon et al., 2020). Enduring barriers to active partnerships and meaningful LE involvement include issues related to remuneration, power imbalances, stigma and discrimination, and a reluctance of educators to work collaboratively with people who have LE (Bennett-Weston et al., 2024; Brand et al., 2023; Happell et al., 2019, 2021).

Simulation-based education (SBE) should include authentic ‘real world’ lived experience to improve the delivery of safe, high quality, consumer-focused healthcare (Brand et al., 2024). However, most SBE is designed by health professions educators who draw on their own clinical knowledge and experience, but do not necessarily have LE of the topic themselves (Nestel et al., 2008). Simulation-based education as a pedagogical approach aims to emulate clinical practice. One modality of SBE is where actors, referred to as simulated patients, play a scripted role of a person accessing healthcare (Kourgiantakis et al., 2020). This offers learners a lower stake or lower risk human presence to explore how they would respond to potential challenges in their professional roles (Micsinszki et al., 2022). However, without LE involvement, assumptions are made by educators about how the simulated patient would think, feel, or respond to the scenario and the interactions they have with health professionals (Brand et al., 2024). In these cases, actors playing the role of simulated patients rely on pre-prepared scripts based on the educator’s assumptions, along with their own personal assumptions to determine how to respond to learners during the simulation (Snow, 2014). This unidirectional approach maintains the status quo of privileging educator knowledge, and is a missed opportunity for the deep transformative learning that occurs from experiential knowledges of people who access healthcare services (Brand et al., 2024). It also brings into question the authenticity of SBE without the involvement of people with LE.

Examples of lived experience involvement in SBE is emerging in the literature, with studies typically describing the SBE and its impacts on learners (Nazarjuk et al., 2013; Orr et al., 2013; Thomas et al., 2014; Williams et al., 2015). The design and delivery of SBE involves six phases: (1) Preparing, (2) Briefing, (3) Simulation activity, (4) Debriefing and feedback, (5) Reflection, and (6) Evaluation (Battista & Nestel, 2018). Levels of LE involvement vary from no involvement (e.g., the designer of the simulation has used quotes from original research studies) to full partnership (e.g., people with LE and educators work together systematically and strategically across all SBE phases). To our knowledge, a comprehensive evaluation of LE involvement in SBE that specifically explores the level of partnership and extent of LE involvement in each phase has not yet been reported. This is notwithstanding calls for guidance on how to best involve LE in the design and delivery of SBE in the absence of published guidelines (Chianáin et al., 2021).

A preliminary search of Ovid MEDLINE, the Cochrane Database of Systematic Reviews, JBI Evidence Synthesis and Open Science Framework was conducted to identify LE involvement in health professions education. There are two published scoping reviews on lived experience involvement in tertiary nursing, midwifery and allied health professions education (Soon et al., 2020; Tørring & Pedersen, 2022). Both reviews do not include SBE. Two scoping reviews have focused on how illness experiences inform simulated patient’s encounters in HPE (Chianáin et al., 2021), and working with older adults as simulated participants (Smith et al., 2023), but did not identify the healthcare disciplines that are harnessing LE in SBE, or determine the levels of partnership. Accordingly, a better understanding of how health professions educators across disciplines are engaging with LE to inform SBE is needed, and findings may offer educators new approaches to involving LE across various SBE phases. It is therefore timely and critical to map what is known about LE service user involvement in SBE for HPE with scoping reviews being useful for mapping bodies of literature that have not yet been comprehensively reviewed (Aromataris et al., 2024). The intention of this scoping review is to identify and describe what is known about LE involvement in SBE in order to determine how to best harness these unique experiential knowledges across HPE to better prepare learners and ensure best practice.

## Methodology

This scoping review was conducted in accordance with the Joanna Briggs Institute (JBI) scoping review methodology (Aromataris et al., 2024) and methods recommended by Arksey and O’Malley (2005) and refined by Levac et al. (2010). All authors contributed to and agreed upon the a priori protocol which was registered in Open Science Framework (https://osf.io/zqpc5). Review findings are reported in accordance with the Preferred Reporting Items for Systematic Reviews and Meta-Analyses Extension for Scoping Reviews (PRISMA-ScR) (Tricco et al., 2018).

### Research aim and questions

This scoping review aimed to examine the available literature on the involvement of lived and living experiences of healthcare in health professions simulation-based education at undergraduate, postgraduate, and continuing professional development levels. The review question was: How is lived experience integrated into health professions simulation-based education?

The review sub-questions were:

1. Which health professions disciplines are including lived experiences in simulation-based education and what are the topics that feature lived experience?
2. How is lived experience being incorporated into each phase of simulation-based education?
3. What is the level of involvement of lived experience in simulation-based education for health professionals?

### Eligibility criteria

Studies were included if they met our defined criteria for participants, concept, and context (PCC). The *participants* included anyone who had participated in SBE for HPE where lived experience had informed one or more simulation phases (Table 1) within all health professions disciplines. This included learners, educators, and people with LE. The terms ‘patient’, ‘consumer’, ‘service user’, ‘client’, and ‘carer’, are often used interchangeably to refer to people who have LE of accessing healthcare. There is no consensus on how and when these terms should be used (Fossey et al., 2023). To define LE, we used Brand et al’s (2023, p1) definition, of “people with knowledge and wisdom gained through lived (and living) experience of a health condition, disability, circumstance (e.g. homelessness), or [priority population] e.g., as defined by culture, race, gender, sexuality, class etc.” Acknowledging that most people will access healthcare during their lives, we further defined LE as a person with unique experiential knowledge of the content matter that is being taught in SBE. Therefore, all unique experiences of health and accessing healthcare services were considered without limitation. We excluded studies if the topic of the SBE was not something that people can describe their LE of. For example, we excluded the study by Boukouvalas et al. (2018) which described a simulation where “people with a lived experience of mental illness” acted as a simulated patient experiencing a mental health crisis, including possible suicidal ideation. People with a lived experience of mental illness is a very broad term and it was not identified whether these people had ever actually experienced crisis or suicidal ideation in real life.

The *concept* to be examined was LE in health professions SBE. We defined health professions education as education targeted at ensuring quality health care, and includes, but is not limited to, medicine, nursing, midwifery, paramedicine, and allied health. To define allied health, we used the Allied Health Professions Australia (2024) definition: “health professionals that are not part of the medical, dental or nursing professions. They are university qualified practitioners with specialised expertise in preventing, diagnosing and treating a range of conditions and illnesses” (para 3).

For the purpose of this review, SBE included all simulation modalities (e.g., part task trainer, simulated/standardised patient, screen-based simulation, or virtual reality) and types (e.g., low, medium, and high fidelity). Simulation-based assessment (e.g., Objective Structured Clinical Exam) was excluded as the aim is to evaluate learner performance, rather than providing an environment to learn through skills practice. Furthermore, assessments are often connected to competencies leaving little flexibility to incorporate LE. To be eligible for inclusion, studies needed to explicitly illustrate LE inclusion in at least one of the six phases of the design and delivery of SBE (Battista & Nestel, 2018) (Table 1).

**Table 1:** Six phases of a simulation program

Phase	Description of activities
Preparing	Activities required to design and prepare the simulation event e.g. choosing the topic, writing learning outcomes, developing the scenario, anticipating challenges for learners.
Briefing	Activities required to ensure learners and SPs know what to expect from the simulation e.g. reviewing learning objectives; outlining how the sim will progress including explanation of the simulation phases and individuals’ responsibilities in each; outlining plans for debriefing and feedback processes.
Simulation activity	Activities required to facilitate learner engagement in the simulation e.g. managing activities, observing learners and identifying feedback for debriefing, taking on role of simulated patient
Debriefing and feedback	Facilitating developmental conversations immediately after simulated activity e.g. facilitator exploring learner feelings, addressing goals and learning objectives, seek other perspectives, summarizing, and affirming positive behaviours. Feedback may be given by the facilitator or SP
Reflecting	Learners are encouraged to make sense of the simulation in the light of their own past and anticipated future experiences.
Evaluation	All participants determine the success and limitations of the simulation in meeting learning outcomes. This can be measured using qualitative and quantitative methods

(Source: Battista & Nestel, 2018)

Using a modified version of Tew et al.’s (2004) *Ladder of Involvement*, we rated the level of partnership in SBE for health professions (Table 2). This ladder has five stages of lived experience involvement.

**Table 2:** Ladder of lived experience involvement in SBE for health professions education

Level 1 No involvement	Stories were collected for purposes other than the simulation e.g. the designer of the simulation has used quotes from original research papers
Level 2 Limited involvement	Inclusion of lived experience in the form of “storytelling,” or in the role of simulated patient. No opportunity to participate in shaping the design or delivery of SBE.
Level 3 Growing involvement	Inclusion of lived experience in two or more of the following areas: preparing, briefing, simulating, debriefing and feedback, reflecting, evaluating. However, lived experience is not represented in key education decisions (e.g. learning outcomes). Equitable payment is made that aligns with how others are paid for working on the same or similar projects. No support, supervision or training in HPE available
Level 4 Collaboration	Lived experience is included in all matters of simulation design and delivery and is involved as full team members in at least 3 areas of the simulation program: preparing, briefing, simulating, debriefing and feedback, reflecting, evaluating
Level 5 Partnership	Lived experience and teaching staff work together systematically and strategically across all areas and this is underpinned by an explicit statement. All key decisions are made in reciprocal or equal partnership. People with lived experience are employed as lecturers on secure or long-term contracts, unless they have chosen to volunteer or be employed on a casual basis.

Studies examining LE in any phase of health professions SBE were considered. This included studies conducted in any geographic location, across all settings where HPE takes place (e.g., hospital or university), and across all education levels (e.g., pre-registration, post-registration, continuing professional development), and modes of delivery (e.g., face-to-face, online).

### Types of sources

This scoping review considered all primary research studies that described a SBE intervention. Grey literature sources (e.g., theses, and dissertations) were also included to ensure that LE in simulation-based HPE was comprehensively captured.

### Search strategy

A three-step search strategy was conducted in March 2024 in consultation with a university librarian. First, an initial search of the Cumulative Index of Nursing and Allied Health Literature (CINAHL) Complete and Ovid MEDLINE databases was conducted to identify relevant articles, keywords, and search terms to inform the full search strategy for Ovid MEDLINE (Supplementary 1). The search strategy was adapted to each individual database. Second, a detailed search for peer reviewed sources was undertaken in CINAHL Complete, Scopus, ERIC, Ovid MEDLINE, and PsycINFO. ProQuest Dissertations and Theses Global Database was searched for grey literature. Third, the reference lists of the studies selected for review were examined for further relevant literature. Studies published in languages other than English were excluded due to limitations in resources to engage quality translation services. We included studies published from January 2004 to March 2024 as this date coincides with the introduction of the ladder of involvement (Tew et al., 2004), which offered a way to categorise involvement of people with LE in HPE.

### Evidence selection

**Fig. 1:**
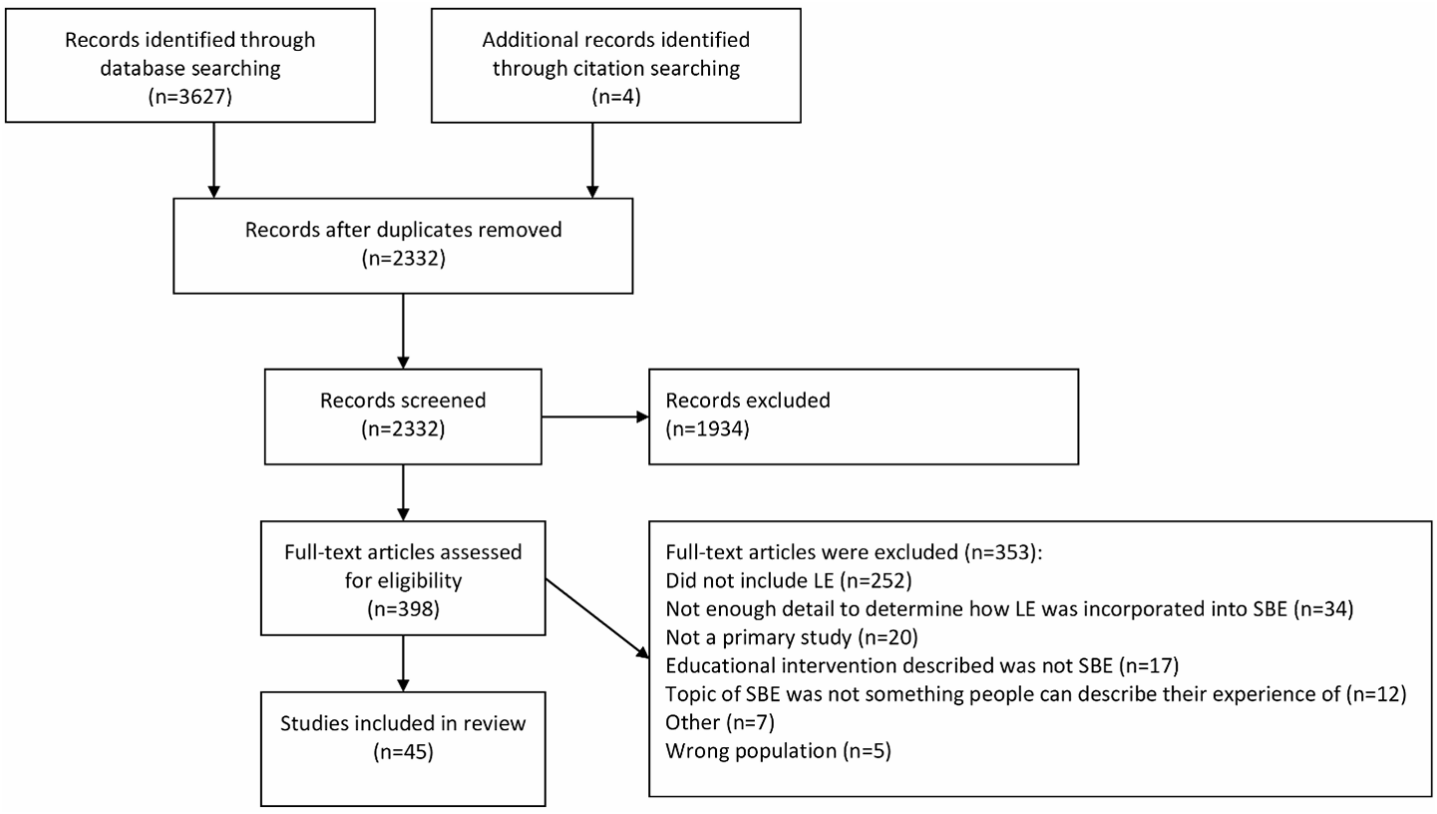
Flow chart of literature search

An initial database search using key search terms identified 3627 records which were collated and imported into Covidence (Veritas Health Innovation). After duplicates were removed, there were 2328 records that were eligible for title and abstract screening. The lead author (RM) screened all titles and abstracts, and secondary screening was conducted by another reviewer (JB, SS, NP, GB). Conflicts were resolved through team discussions. Of the screened studies, 1934 were excluded as they did not meet the inclusion criteria, leaving 398 studies for full-text screening. A further 353 studies were excluded following an independent assessment by two reviewers. Four additional eligible records were identified via hand reference list searches. A total of 45 studies were eligible for inclusion (Figure 1).

### Data extraction

A data extraction tool was created drawing on the diverse knowledge and expertise of the research team and the literature, and incorporating both our modified version of the ladder of involvement (Tew et al., 2004), and six phases of SBE (Battista & Nestel, 2018) (Supplementary 1). The data extraction tool was independently piloted by two reviewers, with a total of 10 (22%) of the included articles undergoing pilot data extraction. After independent pilot extraction all team members met to provide feedback on the tool. No modifications were required. The lead author (RM) independently extracted data from all included studies. Two-thirds (67%) of the included studies were subject to a second independent extraction, with all team members being involved in this process. Reviewers met to enable discussion about discrepancies, however there was no significant discrepancies, and consensus was achieved on the final data to be extracted.

### Data analysis and presentation

All data extracted in Covidence were exported to Microsoft Excel (version 1808) and charted using the built-in data analysis functions to enable visualisation of the answer to the first three review questions: (1) The health professions involving lived experience and the topics commonly featuring lived experience, (2) How lived experience is incorporated into each phase of SBE, and (3) Level of involvement of lived experience in SBE.

Data for question 1 was explicitly stated in the included studies and therefore required no interpretation from authors. When extracting data for question 2, there were studies that did not explicitly identify LE involvement using the six phases of a simulation program (Battista & Nestel, 2018). Therefore, a description of the activities of the six phases of a simulation program (see Table 1) was used to guide extraction. Similarly, for question 3, studies did not explicitly state the level of LE involvement. Reviewers determined the level of LE involvement using pre-determined criteria built into the extraction tool (see Supplementary 2 and Supplementary 3).

## Results

Included studies were published between 2004 and 2024. The geographical spread of included studies was as follows: USA (16), Australia (11), United Kingdom (8), Canada (7), Jordan (1), Netherlands (1), and Switzerland (1). Studies comprised mixed methods (19), qualitative (17), and quantitative (9). Forty-three included studies were peer reviewed, and only two were from grey literature (theses). The majority of participants in the included studies were learners, and most studies reported on the design, implementation and evaluation of a SBE intervention. While LE was identified in all of the included studies, detailing LE involvement was not the aim of the majority of these studies.

### Q1

Which health professions disciplines are including lived experiences in simulation-based education, and what are the topics that feature lived experience?

Studies were from medicine (16), and nursing (14), medicine and nursing combined (2), nursing, medicine and allied health combined (6), pharmacy (2), clinical exercise physiology (1), psychology (1), social work (1), radiation therapy (1) and one study did not identify who their simulation resource was developed for. Lived experience involvement in SBE mostly occurred in undergraduate (30), followed by post-graduate 6) and continued professional development (6). There were (3) studies that reported on SBE in a combination of courses (Table 3).

**Table 3:** Characteristics of included studies

Author, year, title	Country	Grey/Published	Study design	Study Participants (participated in SBE as learner, educator, lived experience)
Rutledge et al. (2004). Using Standardized Patients to Teach and Evaluate Nurse Practitioner Students on Cultural Competency	USA	Published	Mixed methods	Learner
Shane (2006). Technology based pediatric clinical case scenarios for nursing students	USA	Grey (thesis)	Qualitative	Learner
Baer et al. (2008) Breaking Bad News: Use of Cancer Survivors in Role-Playing Exercises	USA	Published	Mixed methods	Learner
Bokken et al. (2010). The case of “Miss Jacobs”: adolescent simulated patients and the quality of their role playing, feedback, and personal impact.	Netherlands	Published	Quantitative	Learner; Educator; Lived experience
Smith et al. (2011). Cultural Competence Clinic: An Online, Interactive, Simulation for Working Effectively With Arab American Muslim Patients	USA	Published	Quantitative	Learner
Orr et al. (2013). The distress of voice-hearing: The use of simulation for awareness, understanding and communication skill development in undergraduate nursing education	Australia	Published	Qualitative	Learner
Nazarjuk et al. (2013). Involving service users in student education	United Kingdom	Published	Mixed method	Learner
Basheti (2014). The Effect of Using Simulation for Training Pharmacy Students on Correct Device Technique	Jordan	Published	Mixed method	Learner
Thomas et al. (2014). Standardised patients with intellectual disabilities in training tomorrow’s doctors.	United Kingdom	Published	Mixed method	Learner
Smeltzer et al. (2015). Persons with Disability: Their Experiences as Standardized Patients in an Undergraduate Nursing Program	USA	Published	Qualitative	Lived experience
Williams et al. (2015). Simulation of cardiac emergencies with real patients	United Kingdom	Published	Quantitative	Learner
Cahill et al. (2015). ‘I wouldn’t get that feedback from anywhere else’: Learning partnerships and the use of high school students as simulated patients to enhance medical student’s communication skills	Australia	Published	Mixed methods	Learner; Lived experience
Horstmanshof et al. (2016). Clinical exercise physiology students learning with older adults: an innovative simulation-based education programme	Australia	Published	Qualitative	Learner; Educator; Lived experience
Min-Yu Lau et al. (2016) Cultural respect encompassing simulation training: being heard about health through broadband	Australia	Published	Mixed methods	Learner
Watkins et al. (2016) Improving healthcare for people with intellectual disabilities: The development of an evidence-based teaching programme.	United Kingdom	Published	Quantitative	Learner
Weideman et al. (2016) Strengthening cultural competence in prenatal care with a virtual community: building capacity through collaboration.	USA	Published	Mixed method	Learner
Ali et al. (2017). Innovative curriculum for second-year Harvard-MIT medical students: practicing communication skills with volunteer patients	USA	Published	Mixed methods	Learner
Attoe et al. (2017) Actors with intellectual disabilities in mental health simulation training	United Kingdom	Published	Qualitative	Lived experience
Corr et al. (2017). Culturally Safe Practices in the Co-creation of Medical Education Curriculum with Indigenous Animators: Outcomes From an Indigenous Learning Circle	United Kingdom	Published	Qualitative	Learner
Thompson et al. (2017). Older people’s views and experiences of engagement in standardised patient simulation.	United Kingdom	Published	Qualitative	Lived experience
Mathew et al. (2017). Designing a Virtual Simulation Case for Cultural Competence Using a Community-Based Participatory Research Approach	USA	Published	Qualitative	Learner
Cosper et al. (2018). The Impact of Patient and Family Advisors on Critical Care Nurses’ Empathy.	USA	Published	Quantitative	Learner
Riches et al. (2019). Impact of an auditory hallucinations simulation on trainee and newly qualified clinical psychologists: A mixed methods cross sectional study.	United Kingdom	Published	Mixed methods	Learner
McCave et al. (2019). Promoting Affirmative Transgender Health Care Practice Within Hospitals: An IPE Standardized Patient Simulation for Graduate Health Care Learners.	USA	Peer reviewed	Quantitative	Learner
Dugmore et al. (2020). Interpreting the value of feedback: Older adult voices in nursing education.	Australia	Peer reviewed	Qualitative	Lived experience
Hartman et al. (2020). An Authentic Poverty Simulation for Health Care Profession Students Using Community Volunteers Experiencing Poverty.	USA	Peer reviewed	Mixed methods	Learner
Kipang (2022). Retooling Social Work Education: New Applications for Collaborative Knowledge Creation, Experiential Learning and Engagement of Experts by Experience	Canada	Grey (thesis)	Qualitative	Learner, educator, lived experience
Maar et al. (2020). Co-creating Simulated Cultural Communication Scenarios with Indigenous Animators: An Evaluation of Innovative Clinical Cultural Safety Curriculum	Canada	Peer reviewed	Mixed methods	Learner; educator
Ozkara San (2020). The influence of the oncology focused transgender simulated patient simulation on nursing students’ cultural competence development.	USA	Peer reviewed	Quantitative	Learner
Palmaria et al. (2020). Learning From Cancer Survivors as Standardized Patients: Radiation Therapy Students’ Perspective.	Canada	Peer reviewed	Qualitative	Learner
Symon, et al. (2021). Practical reflections on a collaboration with healthcare consumers on the development of a simulation.	Australia	Peer reviewed	Qualitative	Other
Takeuchi et al. (2021). ‘Demystifying’ the encounter with adolescent patients: a qualitative study on medical students’ experiences and perspectives during training with adolescent simulated patients.	Switzerland	Peer reviewed	Qualitative	Learner
Tyerman et al. (2021). LGBTQI2S Virtual Simulation: Lessons Learned Using Actors With Lived Experience.	Canada	Peer reviewed	Qualitative	Educator
Garvey et al. (2022). Enhancing Cultural Capabilities Amongst Health Professions Students: A Pilot Study of Interprofessional Tag Team Simulation.	Australia	Peer reviewed	Mixed methods	Learner
Hersh et al. (2022). An Effective Gender-Affirming Care and Hormone Prescribing Standardized Patient Case for Residents.	USA	Peer reviewed	Quantitative	Learner; Educator
Kreines et al. (2022). Training clinicians in culturally relevant care: a curriculum to improve knowledge and comfort with the transgender and gender diverse population	USA	Peer reviewed	Mixed methods	Learner; Lived experience
Maar et al. (2022). Teaching Culturally Safe Care in Simulated Cultural Communication Scenarios During the COVID-19 Pandemic: Virtual Visits with Indigenous Animators.	Canada	Peer reviewed	Mixed methods	Learner; Educator
Sauve et al. (2022). Stand Up for Indigenous Health: A Simulation to Educate Residents About the Social Determinants of Health Faced by Indigenous Peoples in Canada.	Canada	Peer reviewed	Quantitative	Learner
West et al. (2022). Indigenous-led First Peoples health interprofessional and simulation-based learning innovations: mixed methods study of nursing academics’ experience of working in partnership.	Australia	Peer reviewed	Mixed methods	Educator
Bessette et al. (2023). Culturally Safe Practices in the Co-creation of Medical Education Curriculum with Indigenous Animators: Outcomes From an Indigenous Learning Circle.	Canada	Peer reviewed	Qualitative	Lived experience
Pilnick et al. (2023). Conversation Analysis Based Simulation (CABS): A method for improving communication skills training for healthcare practitioners.	United Kingdom	Peer reviewed	Mixed methods	Learner; Educator; Lived experience
Ung et al. (2023). Simulated psychosis care role-plays for pharmacy curricula: a qualitative exploration of student experiences	Australia	Peer reviewed	Qualitative	Learner
Tjia et al. (2023). Using Simulation-Based Learning with Standardized Patients (SP) in an Implicit Bias Mitigation Clinician Training Program.	USA	Peer reviewed	Mixed methods	Learner; Educator; Lived experience
Whited et al. (2024). Promoting communication between paediatric nurse practitioner students and patients with language barriers utilizing an innovative simulation scenario.	USA	Peer reviewed	Mixed methods	Learner
Brand et al. (2024). ‘You don’t see what I see’: Co-designing simulation to uncover and address cognitive bias in healthcare.	Australia	Peer reviewed	Qualitative	Educator; Lived experience

Being culturally and linguistically diverse was the lived experience most commonly included in SBE, with this experience being incorporated into 13 simulations, including preparing learners for culturally-safe communication (Bessette et al., 2023; Maar et al., 2020; Whited et al., 2024), cultural safety and competency (Garvey et al., 2022; Maar et al., 2020, 2022; Mathew et al., 2017; Min-Yu Lau et al., 2016; Rutledge et al., 2004; Smith & Silk, 2011; Weideman et al., 2016; Whited et al., 2024), mitigating bias in clinician-patient encounters (Tjia et al., 2023), and social determinants of health faced by Indigenous peoples of Canada (Sauve et al., 2022).

Five studies mentioned the involvement of LE of disability, with four simulations focussing on providing care for people living with disability (Attoe et al., 2017; Nazarjuk et al., 2013; Smeltzer et al., 2015; Thomas et al., 2014) and one aimed at improving learner attitudes toward people with intellectual disabilities (Watkins & Colgate, 2016). There were also five studies that mentioned involvement of members of the LGBTQI + community, four described the inclusion of people who are transgender and receiving care (Hersh et al., 2022; Kreines et al., 2022; McCave et al., 2019; Ozkara San, 2020) and one described involvement from the broader LGBTQI + community in a sexual orientation and gender identity nursing education toolkit that included simulation (Tyerman et al., 2021). Four studies included the involvement of adolescents with the focus of the simulations being on communication (Bokken et al., 2010; Cahill et al., 2015; Kipang, 2022; Takeuchi et al., 2021). Four studies mentioned LE of cancer or caring for a child with cancer, there was a communication component in all four studies (Baer et al., 2008; Corr et al., 2017; Palmaria et al., 2020; Symon et al., 2021), with two of the studies also focussing on technical skills (Palmaria et al., 2020; Symon et al., 2021).

**Fig. 2:**
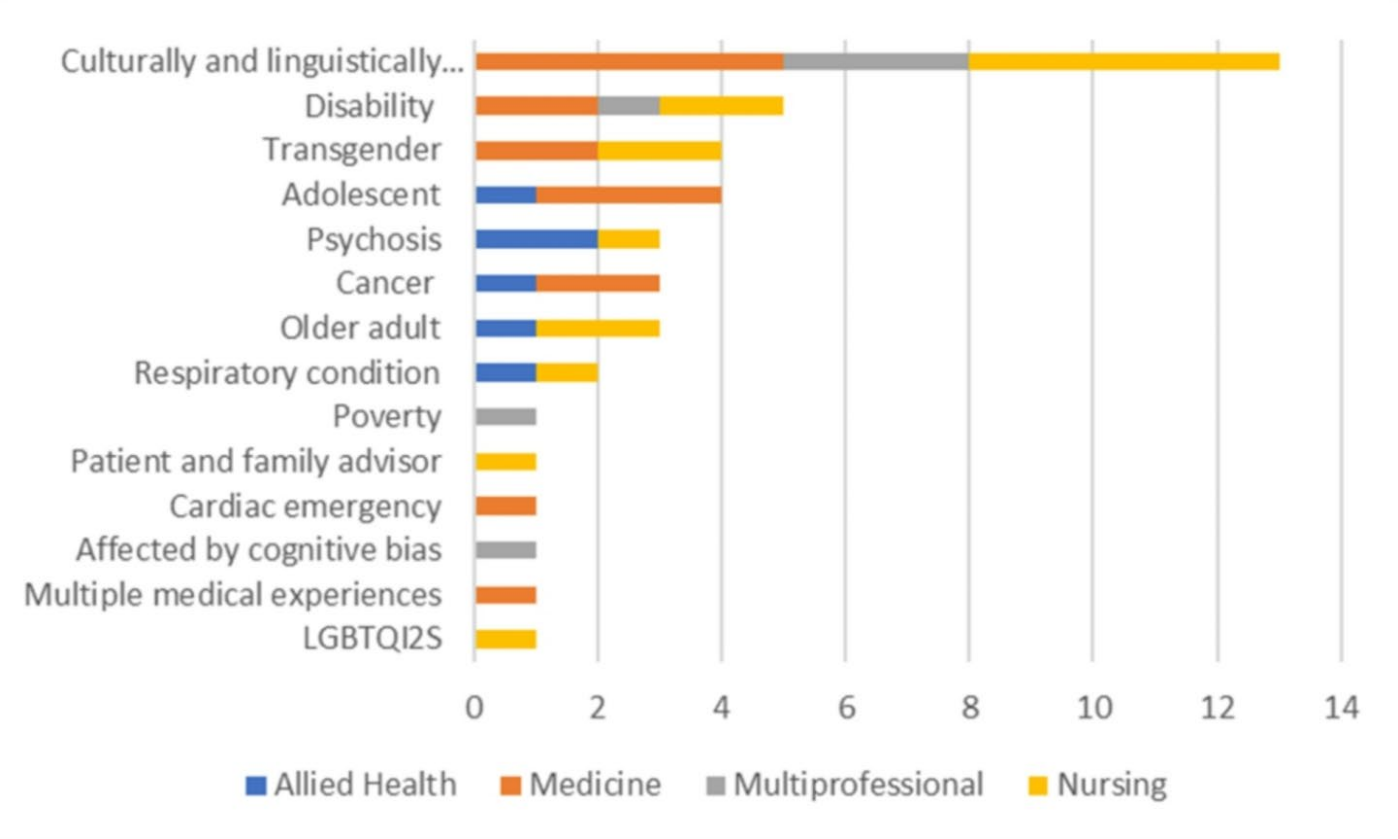
Topics that include lived experience in SBE by health professions discipline

There were three studies describing the inclusion of LE of psychosis in SBE to enhance learners’ capacity to understand and communicate with people experiencing psychosis (Orr et al., 2013; Riches et al., 2019; Ung et al., 2023), and three studies that mentioned involvement of older adults to enhance learner’s communication with older adults (Dugmore et al., 2020; Horstmanshof et al., 2016; Thompson et al., 2017). Other experiences represented in the included studies were respiratory conditions (Basheti, 2014; Shane, 2006), recent cardiac emergencies (Williams et al., 2015), being the carer of a person living with dementia (Pilnick et al., 2023), living in poverty (Hartman et al., 2020) and being a healthcare service consumer and/or carer who has been involved in, or affected by, a healthcare interaction involving cognitive bias (Brand et al., 2024) (see Fig. 2).

### Q2

How is lived experience being incorporated into each phase of simulation-based education?

### The six phases of simulation-based education

**Fig. 3:**
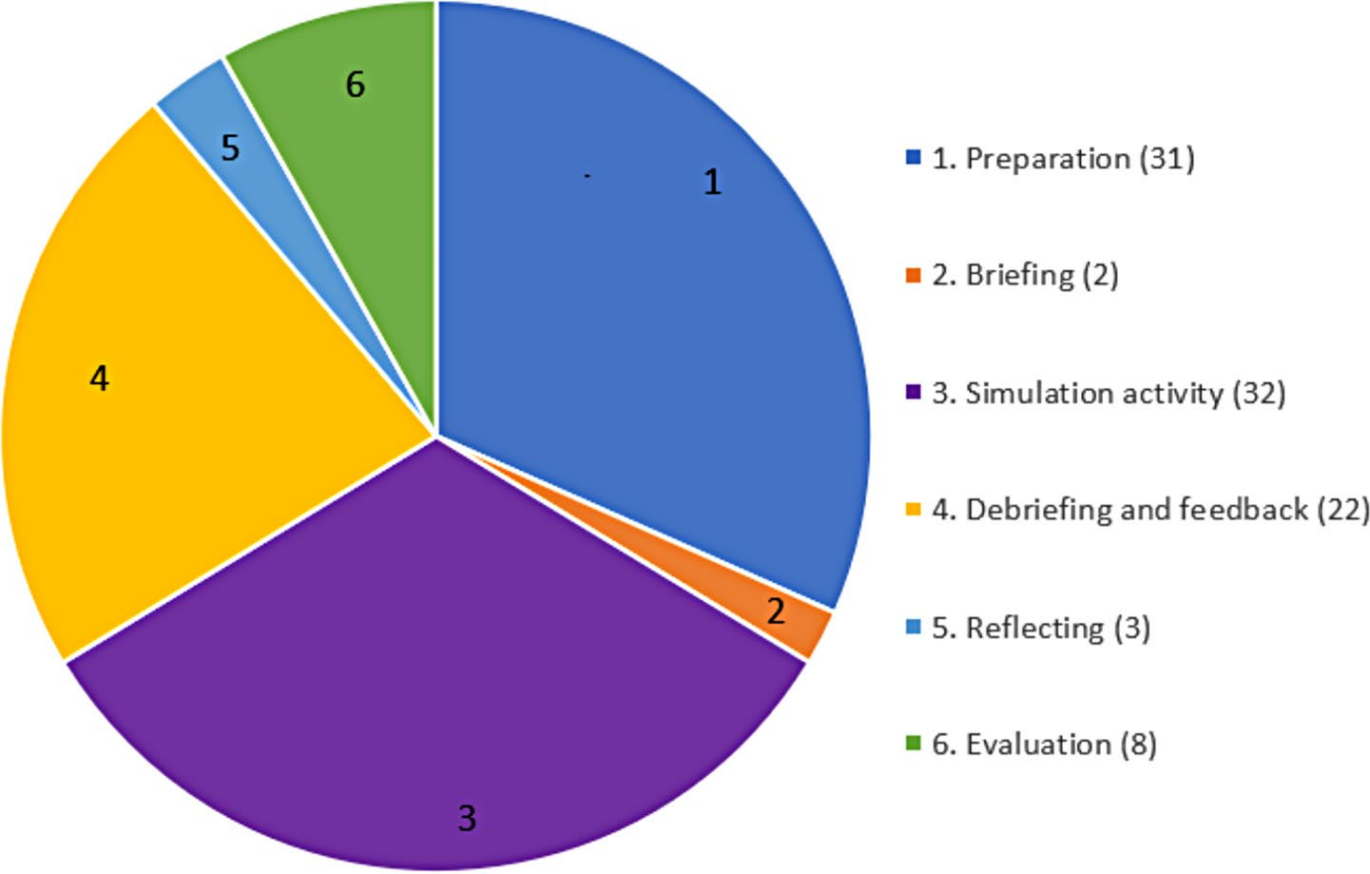
Lived experience involvement in phases of SBE

Lived experience involvement described in each study was mapped to each stage of the six phases of simulation-based education (Fig. 3).

#### Preparing

Preparing includes all of the activities required to design and prepare the simulation event; e.g., choosing the topic, writing learning outcomes, developing the scenario, and anticipating challenges for learners. Of the 31 studies that included lived experiences in this phase, most described active contribution to the process of developing SBE scenarios and the role of simulated patient (Ali et al., 2017; Attoe et al., 2017; Bessette et al., 2023; Bokken et al., 2010; Cosper et al., 2018; Garvey et al., 2022; Hersh et al., 2022; Kipang, 2022; Kreines et al., 2022; Maar et al., 2020; Mathew et al., 2017; McCave et al., 2019; Min-Yu Lau et al., 2016; Pilnick et al., 2023; Riches et al., 2019; Rutledge et al., 2004; Sauve et al., 2022; Smith & Silk, 2011; Symon et al., 2021; Tjia et al., 2023; Tyerman et al., 2021; Ung et al., 2023; Watkins & Colgate, 2016; Weideman et al., 2016). There were four studies where LE was included in the preparation stage, however the level of involvement appeared to be low. In the study by Baer et al. (2008), the clinical scenario used in the role-play was that of a cancer survivor, however it was not made clear how the cancer survivor’s experiences were integrated. Similarly, in the study by Williams et al. (2015), people who had recently experienced cardiac emergencies were asked to recollect the symptoms during the preparation stage, and during simulation they were asked to pretend these symptoms were happening. In the study by Corr et al. (2017), a person with lived experience gave permission for an image of their newly diagnosed melanoma to be turned into a transfer tattoo, but no further contribution to development of the simulation was stated. Shane et al.’s (2006) study reported on use of video footage of unwell children in hospital, however, the children did not actively contribute to development of the simulation.

A small number of studies included LE involvement in contributing to the aims and learning objectives of the SBE (Attoe et al., 2017; Hersh et al., 2022), content validation of educational materials learners can use for self-assessment during the simulation activity (Ung et al., 2023), preparing nurse academics in the use of simulation devices they had designed (Orr et al., 2013), and having full involvement in all aspects of preparing the SBE (Brand et al., 2024; West et al., 2022).

#### Briefing

Briefing occurs immediately prior to the simulation activity and encompasses all of the activities required to ensure learners and simulated patients know what to expect; e.g., reviewing learning objectives; outlining how the scenario will progress including explanation of the simulation phases and individuals’ responsibilities in each; and outlining plans for debriefing and feedback processes. Only two of the included articles described LE involvement in this stage of SBE. In the simulation described by Orr et al. (2013), learner briefing involved listening to a pre-recorded introduction from people with LE of psychosis that provided insight into voice hearing as well as an overview of the workshop. The simulation in the study by Cosper et al. (2018) also included an introduction from people with LE, however this introduction was more of an introduction to their experiences rather than what learners should expect or learn from the simulation.

#### Simulation activity

Simulation activity refers to the actual simulated scenario and includes all of the activities required to facilitate learner engagement in the simulation; e.g., managing activities, observing learners and identifying feedback for debriefing, and taking on the role of a simulated patient. Taking on the role of a patient was the area where most LE involvement occurred. Some studies also mentioned LE involvement in observation of skills using a checklist (Baer et al., 2008), circulating and stimulating discussions (Sauve et al., 2022), providing feedback in the moment (Dugmore et al., 2020; Kreines et al., 2022; Rutledge et al., 2004), coaching (Cahill et al., 2015) and being available to support the facilitation of the simulation (West et al., 2022).

There was variation between studies regarding what the role of the simulated patient involved, and various terms were used to describe this role including ‘patient’ (Basheti, 2014), ‘simulated patient’ (Attoe et al., 2017; Bokken et al., 2010; Dugmore et al., 2020), ‘standardised patient’ (McCave et al., 2019) and ‘animators’ (Bessette et al., 2023; Maar et al., 2020, 2022). Some studies described how people with LE were invited to participate as themselves to provide a real person for learners to communicate with and conduct assessments on (Horstmanshof et al., 2016; Nazarjuk et al., 2013), to teach correct technique of medical devices to (Basheti, 2014), or to answer questions (Weideman et al., 2016). In one study, people with LE were asked to act out their own personal LE (Ali et al., 2017). In other studies, people were provided with a scripted role (Attoe et al., 2017; Dugmore et al., 2020; Palmaria et al., 2020; Thomas et al., 2014; Thompson et al., 2017). Some educators supported simulated patients to modify the script according to their own LE (Dugmore et al., 2020; Watkins & Colgate, 2016). There were some studies that described an improvisational role whereby the person was given key elements that need to be highlighted during the simulation, but had the flexibility to improvise the character around those points (Attoe et al., 2017; Watkins & Colgate, 2016), or they had contributed to the development of the role and were able to deliver the role without the constraints of a standardisation approach (Maar et al., 2022).

#### Debriefing and feedback

Debriefing and feedback may be guided by the person with LE in either a facilitator or simulated patient role. It involves facilitating developmental conversations immediately after the simulated activity; e.g., facilitator exploring learner feelings, addressing goals and learning objectives, seeking other perspectives, summarizing, and affirming positive behaviours. There were several examples of people with LE receiving training in feedback provision (Bokken et al., 2010; Cahill et al., 2015; Kipang, 2022; Min-Yu Lau et al., 2016; Takeuchi et al., 2021; Thomas et al., 2014; Thompson et al., 2017) and debriefing (Thompson et al., 2017) and utilising this training to provide feedback to learners. However, those without prior training were also involved in debriefing and feedback with educators (Ali et al., 2017; Kreines et al., 2022; Maar et al., 2020; Palmaria et al., 2020; Smeltzer et al., 2015; Watkins & Colgate, 2016).

While we did not include sharing experiences and answering learner questions about their LE in our definition of debriefing, it is worth noting that following the simulation activity there were some occasions where this occurred. People with LE in Cosper et al. (2018), Hartman et al. (2020), and Baer et al’s., (2008) studies shared their lived experiences of the simulation topic, with those in Baer et al.’s (2008) study also answering learner questions. In the study by Ozkara San (2020), learners had the opportunity to ask questions about the simulated patients’ LE.

#### Reflection

Reflection creates opportunities for learners to make sense of the simulation in the light of their own past and anticipated future experiences. Only two studies reported on LE in the reflection phase. In Ali et al.’s (2017) study people with LE participated in a collective reflection where learners identified key take home points from their learnings that can shape future practice. Bessette et al. (2023) reported on people with LE providing guidance to learners on their upcoming placement.

#### Evaluation

Evaluation of SBE is the measurement and analysis of success and/or limitations of the SBE in meeting the learning outcomes. In the studies by Baer et al. (2008), Cahill et al. (2015), and Horstmanshof et al. (2016), people with LE participated in evaluation by providing data on their experience of the simulation. In the study by Hartman et al. (2020), people with LE had a more active role in evaluation as planning sessions were periodically conducted to seek feedback and improvements on the SBE. Similarly, in the study by Bessette et al. (2023) people with LE determined whether authenticity and cultural safety had been maintained. An even greater level of active involvement in evaluation was reported on by Garvey et al. (2022), West et al. (2022) and Brand et al. (2024) as people with LE collaborated on how the SBE would be evaluated. For example, Garvey et al. (2022) reported on people with LE developing questions to assess learner experience of the simulation, and the evaluation of the simulation described by West et al. (2022) was led by a person with LE.

##### Q3

What is the level of involvement of lived experience in simulation-based education for health care professionals?

**Fig. 4:**
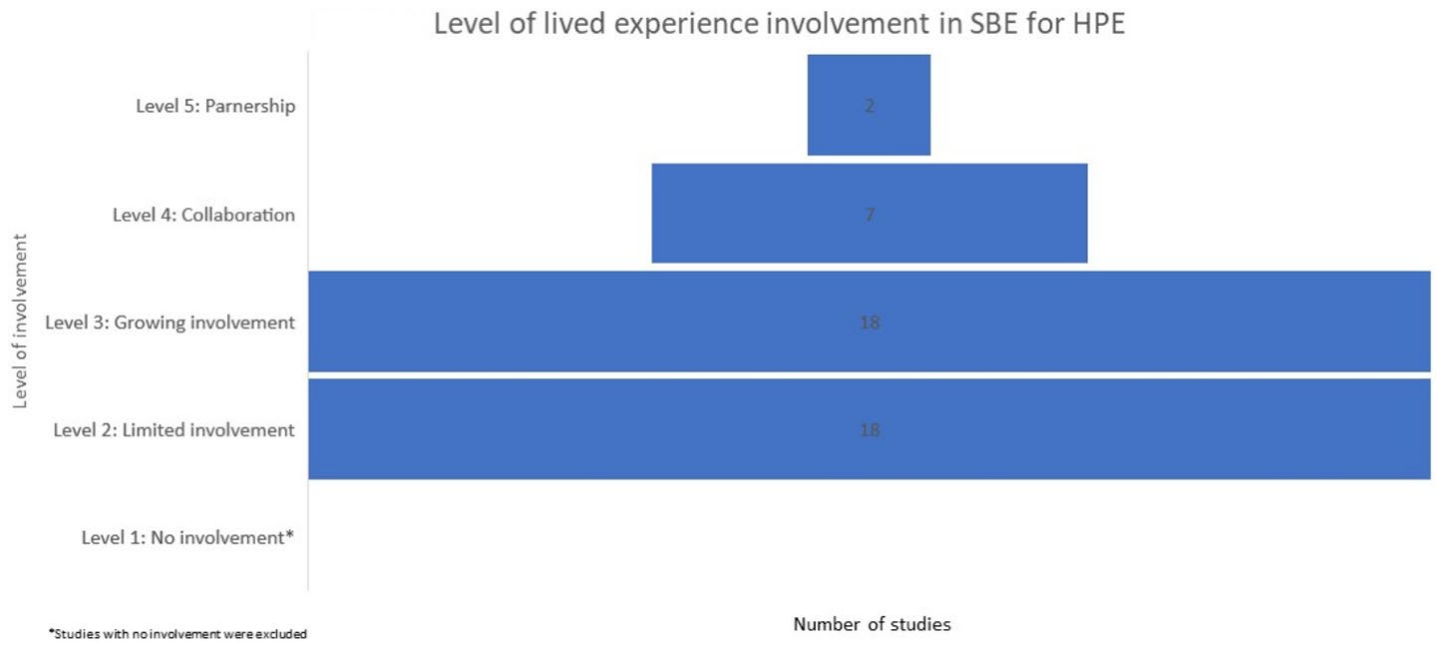
Level of lived experience involvement in SBE for HPE

In determining level of involvement (1: Limited, 2: Growing, 3: Involvement, 4: Collaboration, and 5: Partnership), we considered involvement in all six phases of SBE, rather than involvement in each phase independently. Similar to the broader literature on LE involvement in HPE, involvement in SBE mostly occurred at the lower levels (Fig. 4). There were only two studies that described involvement at level 5: partnership, where people with LE and educators worked together systematically and strategically across all areas, with all key decisions being made in reciprocal or equal partnership (Bessette et al., 2023; West et al., 2022). Bessette et al., (2023) stated that the co-creation process used for their simulation program was built on a longstanding collaborative relationship, and outlined strategies used to ensure authentic co-creation and cultural safety for partners with LE. These strategies focussed on ensuring physical, intellectual/artistic, emotional and spiritual safety for people with LE. The study by West et al. (2022) was the only included study that reported on LE leadership in SBE. This study illustrates how a Professor of Nursing (First People’s health) from an Australian School of Nursing and Midwifery in partnership with the First People’s Health Unit led an initiative to design and deliver a First People’s Health interprofessional and SBE innovation. This innovation included an introductory lecture on First People’s Health delivered by the Professor of Nursing (First People’s health); and a simulated case scenario with compulsory quiz developed by the Professor of Nursing (First People’s health) in collaboration with two First People’s nurse academics. While the simulation was facilitated by non-Indigenous academics, they had received professional development and ongoing support from the Professor, and were encouraged to meet with her as needed.

Seven studies described involvement at level 4 (collaboration) (Ali et al., 2017; Baer et al., 2008; Cosper et al., 2018; Kreines et al., 2022; Maar et al., 2020, 2022; Orr et al., 2013). In these studies, LE were included in all matters of simulation design and delivery and were involved as full team members in at least three areas of the simulation program: preparing, briefing, simulating, debriefing and feedback, reflecting, evaluating. There were 18 included studies at level three (growing involvement, and another 18 at level two (limited involvement) (see Supplement 3). Studies in level three (growing), included the involvement of people with LE in two or more areas of the simulation program, but lived experience was not represented in key education decisions (e.g., learning outcomes). For studies at level two (limited), people with LE had no opportunity to participate in shaping the design or delivery of SBE. Lived experience was only included in the form of “storytelling” or in playing the scripted role of simulated patient.

## Discussion

In this scoping review, we sought to comprehensively review literature related to LE involvement in SBE to understand which health professions disciplines are integrating LE into SBE, the topics they are representing, and how it is being incorporated into the various phases of SBE. We also explored the level of involvement of people with LE that is most common. We only included studies where people had unique experiential knowledge/s of the content matter being taught, therefore review findings provide insight into how LE of specific illnesses and identities are being integrated into SBE for HPE.

Medicine and nursing professions most commonly integrated LE into SBE, typically at the undergraduate level of education. It was beyond the scope of this review to explore the differences between disciplines regarding how inclusion of LE is applied, embraced and supported in SBE. However, given that these differences have been identified in the broader HPE literature (Sinfield et al., 2025) we recommend further research into this topic. People with LE of cultural and linguistic diversity had the greatest amount of inclusion in SBE, with the most common simulation topics being cultural competency and communication. This is not surprising, as there is increasing recognition of the importance of cultural competency in achieving healthcare delivery, and this is increasingly being reflected in health professional licensing legislation (Curtis et al., 2019). This was followed by experiences of living with a disability, identifying as LGBTQI+, being an adolescent, a person living with cancer or caring for one, psychosis, and being an older adult. Experiences of respiratory conditions, cardiac emergencies, cognitive bias, being close to someone who has received care in a hospital, and poverty were also included, but to a lesser degree. The aim of these simulations tended to be on communication with, and care of, individuals with specific illnesses or identities.

Within the broader HPE literature, concerns have been raised about an over-reliance on people with LE for storytelling (LeBlanc-Omstead & Kinsella, 2023). Although this approach can be powerful and represents a shift toward embracing LE as a valid source of knowledge (LeBlanc-Omstead & Kinsella, 2023), some authors have warned that this approach has the potential to be tokenistic and may be a traumatic experience for people with LE (Happell et al., 2015). Soon et al. (2020) recommends including LE in other areas (e.g., curriculum design) to support skill development beyond storytelling, which can be both empowering for people with LE and could potentially lead to increases in their confidence and self-esteem. Findings from our review indicate that SBE provides important opportunities for involvement that extends beyond storytelling, as LE is being harnessed in each simulation phase using the stages defined by Battista and Nestel (2018). However, while SBE does provide diverse opportunities for moving beyond storytelling, we caution that LE involvement in HPE has been critiqued for too often leading to exploitation, tokenism and co-optation (Agrawal et al., 2025), and there is potential for this to occur when involving LE at each stage.

Most LE involvement occurred during the simulation activity phase, with LE being harnessed in various ways such as observing learner skills, stimulating discussions, providing feedback, coaching, and supporting the facilitation. However, the most common role taken on in this phase was providing a real person in real time for learners to interact with. Similar to Chianáin et al’s (2021) scoping review of how illness experiences inform simulated participants’ encounters in HPE, we found inconsistent use of terminology to describe the role of the person representing the illness experience (e.g. simulated patient, standardised patient, animator). Furthermore, we found that the requirements, scope, and degree of autonomy associated with this role differed between the included studies. It has previously been recommended by Plaksin et al. (2016) that all studies reporting on “simulated patient” and “standardised patient” involvement should define how they use the term due to varying definitions or terms being used interchangeably. We agree with these recommendations; however, we argue that there is also a need to prioritise future research aimed at standardising terminology used to describe the variations and requirements of these roles across health professions disciplines and learning contexts.

The preparation stage includes activities crucial in shaping subsequent phases such as choosing the simulation topic, creating learning outcomes, anticipating learner challenges and developing the simulation scenario and simulated patient roles. Calls have long been made to involve LE in role and scenario development (Chianáin et al., 2021; Nestel et al., 2008). Despite little guidance on *how* this should be approached (Chianáin et al., 2021), findings from our review provided several illustrations of this involvement. Conversely there was minimal involvement in choosing the education topic, creating learning outcomes and anticipating learner challenges. To amplify the perspectives and strengthen the voice of people with LE, continued efforts must be made to move them from the periphery to the centre of the education process (Brand & Dart, 2022), including the preparation stages. Our findings suggest that calls for LE involvement in SBE are being answered, however people with LE are not being fully involved in shaping the overall program outcomes. Therefore, in the context of SBE, people with LE are still not at the centre of the education process.

Equal partnership is considered the ultimate goal of HPE involvement initiatives, yet most initiatives are not achieving this (Bennett-Weston et al., 2023). Findings from this review are consistent with previous studies that mapped the literature on patient involvement in HPE and found only a small number of studies that could be considered full partnership (Dijk et al., 2020; Gordon et al., 2020). To determine the level of LE most common in SBE we categorised each study using a modified version of the ladder of involvement (Tew et al., 2004) and found that measures are being taken to increase LE involvement in SBE, for example providing training in providing learners with feedback. However, only two studies achieved the level of full partnership (see Fig. 4), meeting the criteria of working together systematically and strategically across the entire SBE intervention and making all key decisions in reciprocal or equal partnership. It is worth noting here that in both of these studies educators leveraged existing partnerships with LE organisations, and in one of these studies the Professor leading the simulation had lived experience of the topic being taught as they were a Professor of Nursing (First People’s health).

This notion of patient partnerships in HPE only being achievable at the highest levels of involvement was recently challenged by Bennett-Weston et al. (2024), whose qualitative study into patient, educator and learner understandings of partnerships in HPE found equal partnerships are neither feasible or desirable all the time. Rather, they argue that true partnership means valuing patients for their contributions at any level of involvement with tangible approaches to being valued including renumeration; positive feedback; feedback on the impact of their involvement on students; and explicit markers of university recognition such as name badges and email addresses, and access to training. Furthermore, Bennett-Weston et al., (2024) caution on use of frameworks such as the *Spectrum of Involvement* (Towle et al., 2010), a framework based on the *Ladder of Involvement* (Tew et al., 2004), as partnership is only achievable at the highest level. Framing patient partnerships around highest level of involvement risks downplaying the value of patients’ contributions in less prominent roles and creates a sense of failure for those who may not be able to support equal involvement in all aspects of curriculum. In our findings, we observed examples of active involvement at each phase, however when we considered involvement across the entire curriculum (every phase of SBE) the level of involvement decreased. By measuring partnership in this way, we may have downplayed the value of some LE involvement. Therefore, we argue that meaningful partnership should be defined by the person with LE, and thus should be the aspiration of LE involvement in SBE. The focus should not be on reaching the top level of a ladder or spectrum created by academics. There is currently a gap in research conceptualising what meaningful partnership means in SBE, further research is needed to understand what LE partnership should look like, and how it should be reported and evaluated, from the perspective of those with LE. Furthermore, we encourage future SBE partnerships with people who have LE to continue through co-evaluation and publication.

### Strengths and limitations of this review

A strength of this review is that we undertook a comprehensive review of the literature from seven databases and included grey literature. However, our search was limited to studies published in English. This may have introduced a bias in our results, and we may have missed perspectives outside of non-English speaking countries. Due to lack of conceptual clarity around the term ‘lived experience’ in HPE literature we defined the boundaries of this term as having unique experiential knowledge of the content matter that was being taught in SBE. Therefore, our scoping review does not map all LE involvement in SBE, it only maps involvement where LE aligns with the simulation topic. However, by including all health disciplines at various education levels (e.g. undergraduate, post-graduate and CPD) we have been able to broadly capture literature on LE involvement. Describing LE involvement in SBE was not the aim of most included studies; the aim tended to be on describing the SBE intervention and its impact. In these studies, the description of how LE was incorporated into the SBE was brief. This made it difficult to draw conclusions about the level of involvement, and there may have been greater involvement than what was described in these studies. Finally, we used the six phases of SBE (Battista & Nestel, 2018) to guide our reporting on how LE are being incorporated into each phase of SBE. We acknowledge that this approach may be better suited to the standardised patient modality rather than screen-based simulation, or virtual reality. Furthermore, in measuring the level of partnership we took into consideration how many phases the person with LE was involved in. This meant that a study was only considered full partnership if LE was mentioned in each stage.

## Conclusions

Partnership between educators and people with LE is not common across all phases of SBE design and delivery. Despite recommendations that partnerships with people with LE should be central to the education of current and future medical, nursing and allied health professionals, involvement mostly occurs in only two or three of the phases of SBE. This is not to say that involvement at each individual phase was not a meaningful partnership. Simulation based education presents an opportunity to broaden the scope of LE involvement in HPE, moving beyond one-off guest lectures and storytelling to involvement in all phases of curriculum development. By collating findings of involvement in all six phases, educators from the included health professions disciplines can consider ways to harness LE across all simulation phases. Our findings provide recommendations to be incorporated into guidelines on involving LE in HPE. This is particularly important given that no published guidelines currently exist.

While we do emphasise the importance of LE involvement in SBE for HPE to ensure authenticity, we also acknowledge it may not always be possible to recruit people with LE that aligns with the simulation topic. Leveraging existing partnerships and collaborations and working with local LE organisations may assist to ensure authenticity and prevent educator bias.

## Electronic supplementary material

Below is the link to the electronic supplementary material.

Supplementary Material 1

Supplementary Material 2

Supplementary Material 3

## Data Availability

No datasets were generated or analysed during the current study.
