# Supplementary figures and images for: Harnessing lived experience in health professions simulation-based education: a scoping review
Source: Adv Health Sci Educ Theory Pract. 2025 Apr 24;31(1):59–85. doi: 10.1007/s10459-025-10432-9 (PMC12929333; doi:10.1007/s10459-025-10432-9)

**Appendix 1: Full search strategy for Ovid MEDLINE**


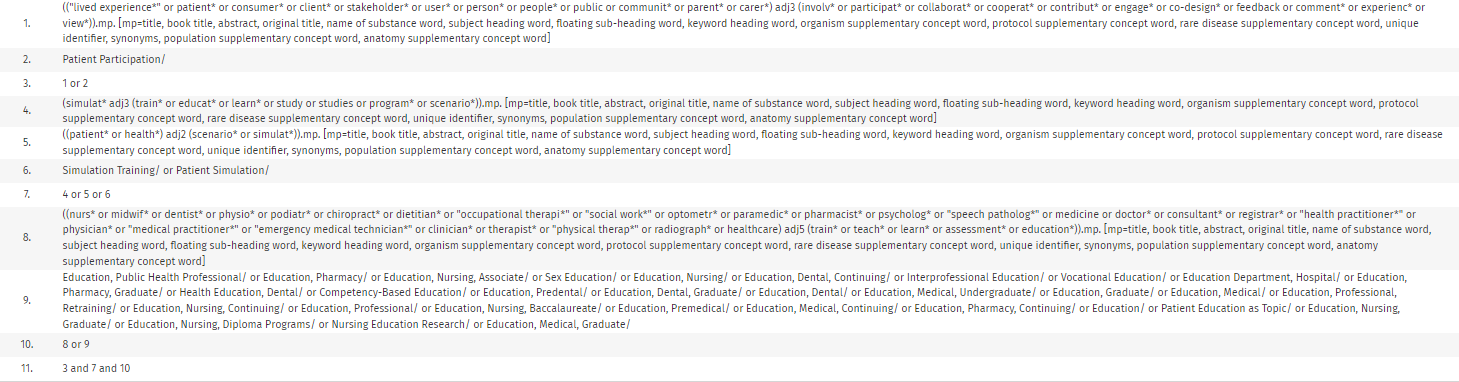

Supplement: Supplementary file 1 — Supplementary Material 1 [file 10459_2025_10432_MOESM1_ESM.docx]
