# Supplementary material for: Harnessing lived experience in health professions simulation-based education: a scoping review
Source: Adv Health Sci Educ Theory Pract. 2025 Apr 24;31(1):59–85. doi: 10.1007/s10459-025-10432-9 (PMC12929333; doi:10.1007/s10459-025-10432-9)
Supplement: Supplementary file 2 — Supplementary Material 2 [file 10459_2025_10432_MOESM2_ESM.docx]

Supplementary 2: Data extraction tool

|  |  | **Bibliographic Information** | | | | **Study/ source description** | | | | | | **Participants** | | |
| --- | --- | --- | --- | --- | --- | --- | --- | --- | --- | --- | --- | --- | --- | --- |
| Data extractor (name) | Article number (as per Covidence) | Author | Primary author email | Year | Title | Country | Setting (hospital, university, online) | Grey/ peer reviewed | Design | Aim | Sample size | Learner Y/N | Educator | Person with lived experience |

| **Concept** | | | | | | | | | | | | | | |
| --- | --- | --- | --- | --- | --- | --- | --- | --- | --- | --- | --- | --- | --- | --- |
| Simulation topic | Description of lived experience (LE) | LE involvement: Preparing (Y/N) | Example of LE Preparing | LE Involvement: Briefing (Y/N) | Example of LE Briefing | LE Involvement: Simulation (Y/N) | Example of LE simulation | LE Involvement: Debriefing & feedback (Y/N) | Example of debriefing and feedback | LE Involvement: Evaluation | Example of LE evaluation | Source of information | Level of involvement in simulation program determined by what is reported (1-5) | Rationale for level |

| **Context** | | **Results** | | | | | **Other** |
| --- | --- | --- | --- | --- | --- | --- | --- |
| Education level (pre or post registration, CPD) | Education discipline | Quantitative results | Qualitative results | Barriers to LE involvement | Enablers to LE involvement | Advice for LE involvement | Other comments |
