# Supplementary material for: Harnessing lived experience in health professions simulation-based education: a scoping review
Source: Adv Health Sci Educ Theory Pract. 2025 Apr 24;31(1):59–85. doi: 10.1007/s10459-025-10432-9 (PMC12929333; doi:10.1007/s10459-025-10432-9)
Supplement: Supplementary file 3 — Supplementary Material 3 [file 10459_2025_10432_MOESM3_ESM.docx]

Supplementary 3: Level of involvement of lived experience in simulation-based education for health professions

| **Level 1: No involvement**  Stories were collected for purposes other than the simulation e.g. the designer of the simulation has used quotes from original research studies | | |
| --- | --- | --- |
| Studies at this level were excluded | | |
| **Level 2: Limited involvement**  No opportunity to participate in shaping the design or delivery of SBE. Inclusion of lived experience in the form of “storytelling,” or in the role of simulated patient. | | |
| Pilnick 2023  Williams 2015  Tjia 2023  Takeuchi 2021  Symon 2021  Smith 2011 | McCave 2019  Mathew 2017  Horstmanshof 2016  Corr 2017  Basheti 2014  Shane 2006 | Rutledge 2004  Nazarjuk 2013  Riches 2019  Ozkara San 2020  Hersh 2022  Whited 2024 |
| **Level 3: Growing involvement**  Inclusion of lived experience in two or more of the following areas: preparing, briefing, simulating, debriefing and feedback, reflecting, evaluating. However, lived experience is not represented in key education decisions (e.g. learning outcomes). Equitable payment is made that aligns with how others are paid for working on the same or similar projects. | | |
| Kipang 2022  Cahill 2015  Ung 2023  Suave 2022  Min-YuLau 2016  Hartman 2020  Brand 2024 | Bokken 2010  Thomas 2014  Watkins 2016  Weideman 2016  Palmaria 2020  Smeltzer 2015 | Tyerman 2021  Dugmore 2020  Garvey 2020  Attoe 2017  Thompson 2017 |
| **Level 4: Collaboration**  Lived experience is included in all matters of simulation design and delivery and is involved as full team members in at least 3 areas of the simulation program: preparing, briefing, simulating, debriefing and feedback, reflecting, evaluating | | |
| Baer 2008  Maar 2020  Ali 2017  Maar 2022  Kreines 2022  Orr 2013  Cosper 2018 | | |
| **Level 5: Partnership**  Lived experience and teaching staff work together systematically and strategically across all areas and this is underpinned by an explicit statement. All key decisions are made in reciprocal or equal partnership. People with lived experience are employed as lecturers on secure or long-term contracts, unless they have chosen to volunteer or be employed on a casual basis. | | |
| Bessette 2023  West 2022 (this is the only study lead by a person with lived experience) | | |
